# Gastrointestinal nematodes and *Fasciola hepatica* in Norwegian cattle herds: a questionnaire to investigate farmers’ perceptions and control strategies

**DOI:** 10.1186/s13028-021-00618-7

**Published:** 2021-12-04

**Authors:** Tonje Opsal, Ingrid Toftaker, Ane Nødtvedt, Lucy Jane Robertson, Kristoffer Relling Tysnes, Ian Woolsey, Lisbeth Hektoen

**Affiliations:** 1 Department of Production Animal Clinical Sciences, Faculty of Veterinary Medicine, Norwegian University of Life Sciences, Universitetstunet 3, 1433 Ås, Norway; 2 Department of Paraclinical Sciences, Faculty of Veterinary Medicine, Norwegian University of Life Sciences, Universitetstunet 3, 1433 Ås, Norway

**Keywords:** Anthelmintic treatment, Grazing, *Ostertagia ostertagi*, Pasture parasites

## Abstract

**Background:**

Pasture management influences the prevalence and impact of the pasture parasites (PP) in cattle herds, which cause production-limiting disease worldwide. Evaluating farmer management strategies is vital when considering sustainable PP control practices. The aim of this questionnaire-based study was to describe the pasture management and control strategies regarding PP in Norwegian beef cattle (BC) and dairy cattle (DC) production systems with a focus on gastrointestinal nematodes (GIN) and *Fasciola hepatica.*

**Results:**

A total of 745 responses from BC (return rate 20.5%) and 1347 responses from DC farmers (30.7%) were included. The mean total pasture time for DC was 4.2 months for first-season grazers and 4.3 months for second-season grazers and cows, while the corresponding finding in BC was 5.4 months. Home pasture was used for most of the pasture period, particularly for first-season grazer dairy heifers (81%), which were also commonly grazed on the same pasture every year (79%). For most farmers it was necessary for grazing areas to be used for cattle for more than one season (77% of BC farmers and 89% of DC farmers). However, changing the pasture during the season was common in both DC (67%) and BC (70%) herds. The majority of DC farmers (60%) stated that they did not consider that they had a problem with PP. Of the remaining 40%, few respondents could specify whether their herds had a problem due to infection by GIN (11%) or liver flukes (12%). Treatment for GIN was performed by 52% of DC and 34% of BC farmers. Diagnostic faecal samples were collected upon suspicion of parasitic disease by 5% of DC and 16% of BC farmers. Veterinarians were stated as a central source of information about parasite management and treatment.

**Conclusions:**

Potential risks for exposure to PP were identified, such as use of the same pasture every year for first-season grazers and frequent use of home pasture. The perception of problems related to PP appeared low. Regular anthelmintic treatment without concurrent use of diagnostic faecal samples seems to be common practice.

**Supplementary Information:**

The online version contains supplementary material available at 10.1186/s13028-021-00618-7.

## Background

Pasture parasite (PP) infections are amongst the most important production-limiting diseases of grazing cattle worldwide. Severe disease can occur, but the major economic impact is due to sub-clinical infections causing reductions in milk yield, growth and fertility [1]. The prevalence and importance of PP vary both between and within countries and are strongly influenced by husbandry practices (e.g., pasture management) and weather conditions [2, 3]. Beef and dairy production are important cornerstones of the Norwegian livestock industry, but, in comparison with many other countries, the production units are small. The median herd size of Norwegian dairy cattle (DC) production units in 2020 is 22 cow years with a range of 1–130, (personal communication Håvard Nørstebø, TINE SA) while the corresponding in beef cattle (BC) production units in 2018 is 15 cow years with a range of 0–264 (personal communication Solveig Bjørnholt, Animalia; Norwegian Meat and Poultry Research Centre). The land suitable for agriculture in Norway is limited, and in areas where crop production is challenging grass-based livestock production is the most efficient way to utilize farmland and uncultivated areas for food production. Studies describing the occurrence and impact on health and production caused by PP infection in Norwegian cattle have been scarce in recent decades. However, studies of gastrointestinal nematodes (GIN) in the 1970s and 1980s found that *Ostertagia ostertagi* and *Cooperia oncophora* were the dominant species in Norwegian cattle, potentially impacting the growth rate of grazing calves [4, 5]. *O. ostertagi*, the brown stomach worm, can cause parasitic gastroenteritis in calves during their first grazing season. Its pathogenic effect is often aggravated by co-infection with *C. oncophora* in the small intestine.

Based on post-mortem registrations from abattoirs, the distribution of the liver fluke *Fasciola hepatica* seems to be focused in southwestern areas of Norway, with positive registrations in 2019 in 6% and 7% of the herds in the southwest counties, Rogaland and Vestland, respectively (personal communication Ragnhild Marit Arnesen Mattilsynet; Norwegian Food Safety Authorities, and Ola Nafstad, Animalia; Norwegian Meat and Poultry Research Centre). The climate in this part of Norway is relatively mild with high precipitation. This is favourable for the parasite life cycle, in which the intermediate host, *Galba truncatula*, thrives in small water bodies on pasture. The impact of *F. hepatica* on production in Norwegian cattle and the routines for anthelmintic treatment have not been studied. However, anecdotal reports from the Norwegian cattle industry suggest increasing concerns regarding the occurrence and management of liver fluke infections.

The extent to which management affects the level of livestock exposure to PP is a frequent focus for research. Several studies have used detection of antibodies in milk or sera (quantified by ELISA and expressed as a corrected optical density ratio, ODR) to evaluate exposure to helminths [6–9]. A study performed in 2006–2007 considered pasture management factors in five countries in north-western Europe [7], including Sweden, a neighbouring country of Norway. Analysis of bulk tank milk (BTM) samples from participating herds indicated that levels of GIN exposure differed between the five countries. Management factors were significantly associated with GIN exposure, suggesting that interventions in pasture management may have a crucial effect in reducing infection levels [7]. A European study modelling the spatial distribution of *F. hepatica*, reported that the prevalence varied significantly between herds in areas with similar climatic conditions, suggesting that pasture management factors are key drivers of infection risk [10].

As subclinical helminth infections in cattle are common, their control may be challenging [11] and relies predominantly on the indiscriminate use of anthelmintic drugs [12]. However, sustainable PP control practices require the use of diagnostic methods to enable informed treatment decisions [11]. Although all classes of anthelmintics are only available by veterinary prescription in the Nordic countries, prophylactic administration of anthelmintic drugs has been identified as an important strategy to control GIN in Swedish cattle production [13]. Based on sales figures from The Norwegian Medicines Agency, there is reason to believe that prophylactic treatment is common in Norway too, as rough estimates indicate that at least 20–25% of first-season grazing cattle in Norway are annually treated with intermittent-release intraruminal boluses containing the benzimidazole oxfendazole (personal communication Knud Torjesen, The Norwegian Medicines Agency). In other countries, intensive use of anthelmintics in cattle has led to the emergence of anthelmintic resistance (AR) [14–16]. To slow down the development of AR there is an urgent need to employ targeted use of anthelmintics [17, 18]. In Norway, AR has been documented in GIN in sheep [19]. AR in PP in cattle has not been investigated.

Because PP management is in the control of the farmer, it is important to comprehend how farmers perceive the challenges associated with PP and how they make their management decisions [20]. Attitudes towards diagnostics, as well as farmers’ perceptions of social pressure of important referents, have been suggested as being major factors positively influencing the adoption intention for diagnostic methods [21]. To understand the impact of PP in Norwegian cattle herds, more knowledge of both beef- and dairy-farmers’ pasture-management and control strategies is needed. Due to the limited focus on PP in Norway, such as a lack of national prevalence studies of *O. ostertagia* and *F. hepatica* relative to studies in other European countries, our current knowledge is limited; this is a disadvantage for farmers, veterinarians, and other animal health workers. Questionnaire-based surveys of Norwegian sheep farmers found that parasite management in sheep was suboptimal [22, 23]. A comparative questionnaire survey regarding pasture use and PP control practices in Norwegian cattle herds has not been performed. This study is part of the project “Sustainable management of pasture parasites in Norwegian beef and dairy cattle (BoviPar)” that aims to provide more knowledge on *F. hepatica* and *O. ostertagi* in Norwegian cattle herds. Other PP such as lungworms, tapeworms, and protozoa have been excluded. Thus, the aim of this questionnaire-based study was to describe farmers’ pasture management and control strategies regarding PP in Norwegian cattle production systems, with emphasis on: (1) general pasture management measures that may influence the prevalence of PP; (2) farmers’ perceptions on the occurrence and importance of *O. ostertagi* and *F. hepatica* in their herds; and (3) treatment strategies employed.

## Methods

### Questionnaires

Dairy cattle (DC) farmers responded to a web-administered questionnaire, using the Questback Online Survey Tool (Questback A/S). The information letter to the farmers described the focus of the questionnaire as being on *F. hepatica* and *O. ostertagi.* Two pilot studies were carried out, one by personal meeting with a small focus group and another by telephone, to ensure precise interpretation of the final questionnaire and evaluate the applicability of the e-mail administration. Modifications were made based on evaluation of the pilot studies. The final questionnaire was distributed by e-mail on 23rd of January 2020 to farmers who were members of TINE SA, the largest dairy cooperative in Norway. A stratified random sampling approach was used. The questionnaire was sent to 4407 dairy farmers grouped by county, skipping every 3rd holding from a list of identification numbers. These represented 58% of the 7599 dairy herds in Norway in 2019 [24]. The list was retrieved from the Norwegian Dairy Herd Recordings System (NDHRS) where approximately 98% of all dairy herds are members [25]. As an incentive to increase the response rate, a gift certificate was offered for one randomly selected respondent. The entire survey required approximately 10 min to complete. Two nudges were sent after 2 and 4 weeks to increase the response rate. The latest response was collected at the end of March 2020.

Data regarding beef cattle (BC) farmers were collected from a questionnaire performed by three undergraduate veterinary students at the Norwegian University of Life Sciences in the summer of 2018. Questions addressed both external and internal parasites of cattle. A pilot study was not performed. This survey was distributed by Animalia, the Norwegian Meat and Poultry Research Centre, using the Enalyzer Survey Solution (Enalyzer A/S). Every member of the Norwegian Beef Herd Recording System registered with an e-mail-address, comprising 3627 beef-farmers in June 2018, received the questionnaire. These represented 67% of the 5427 beef cattle herds in Norway in 2018 [24]. Two nudges were sent to non-responders. The latest response was collected at the end of July 2018.

As the questionnaires to the beef and dairy farmers were not identical, some variables exist for one group only. English translations of the questions are provided in the supplementary data (Additional files 1, 2). The questions were closed ended, with mostly multiple-choice options, and comments were allowed for a small proportion of the questions. The possibility of skipping questions was available. The first set of questions addressed the type of production system (organic/conventional) and the animal species included in the production unit. Information on the timing and duration of the grazing season was requested for every farm. For questions regarding pasture-management practices, farmers were asked to respond to statements using a 5-point Likert-type scale where 1  =  no agreement and 5  =  very high agreement, or they could mark the statements as irrelevant. The subjective experience of problems related to PP was stated on a scale from 0  =  no problem to 4  =  very serious problem. The management and routines for treatment of parasites were key questions, and respondents were routed to different follow-up questions based on their answers. Young animals were defined as animals up to and including 24 months, and adult animals as animals older than 24 months of age. In other questions, the animals were grouped according to whether they were first-season grazers (FSG) or second-season grazers (SSG) and older.

### Data management and statistical analysis

Data were collected and stored according to the General Data Protection Regulation (GDPR) [26]. The questionnaires were checked for completion, and some variables regarding treatment, such as frequency and evaluation of effect, were excluded from the analysis due to a low number of responses restricting interpretation of the results. The information obtained was grouped as follows:

1. Questions about the production unit and details about the farmer (age, total amount of years being a farmer, level of education).
2. Variables describing the nature of the pasture and grazing period.
3. Variables concerning pasture management that could potentially contribute to a high or low parasite burden.
4. Variables describing farmer perceptions of whether PP are a problem in the herd.
5. Variables regarding the frequency and routines for treatment.

The data from the questionnaires were received as excel-files, Excel Office 365 (Microsoft Inc.) and exported to STATA 16 (Stata Statistical Software: Release 16. College Station, TX: StataCorp LP) for cleaning and statistical analyses. Descriptive tables were made for the production unit data, the pasture characteristics and grazing time, farmer perceptions of problems related to PP, and treatment routines related to PP. For categorical variables, counts and proportions were calculated for beef and dairy herds separately. For continuous variables, means and standard deviations (sd) or median and range, were used depending on the frequency distribution of the variable. The geographical distribution of respondents and the population of Norwegian herds were visualized in maps using the quantum geographic information system, QGIS 3.10 (QGIS Development Team, 2020) [27] and presented as the relative frequency (%) in each county. Census population data were retrieved by the Norwegian Agriculture Agency [24]. The pasture management practices in beef and dairy herds were assessed in a stacked bar chart. Regional differences in pasture management practices were investigated for measures directly related to pasture management for liver fluke infection, as these are known to be focused in the south-western areas of the country.

## Results

### The production units and farmer information

The questionnaire design allowed respondents to skip questions, thus many questions were not answered by all the recipients and particularly some routed questions had a low response rate. The number of respondents therefore varied between questions.

#### Dairy cattle respondents

**Fig. 1:**
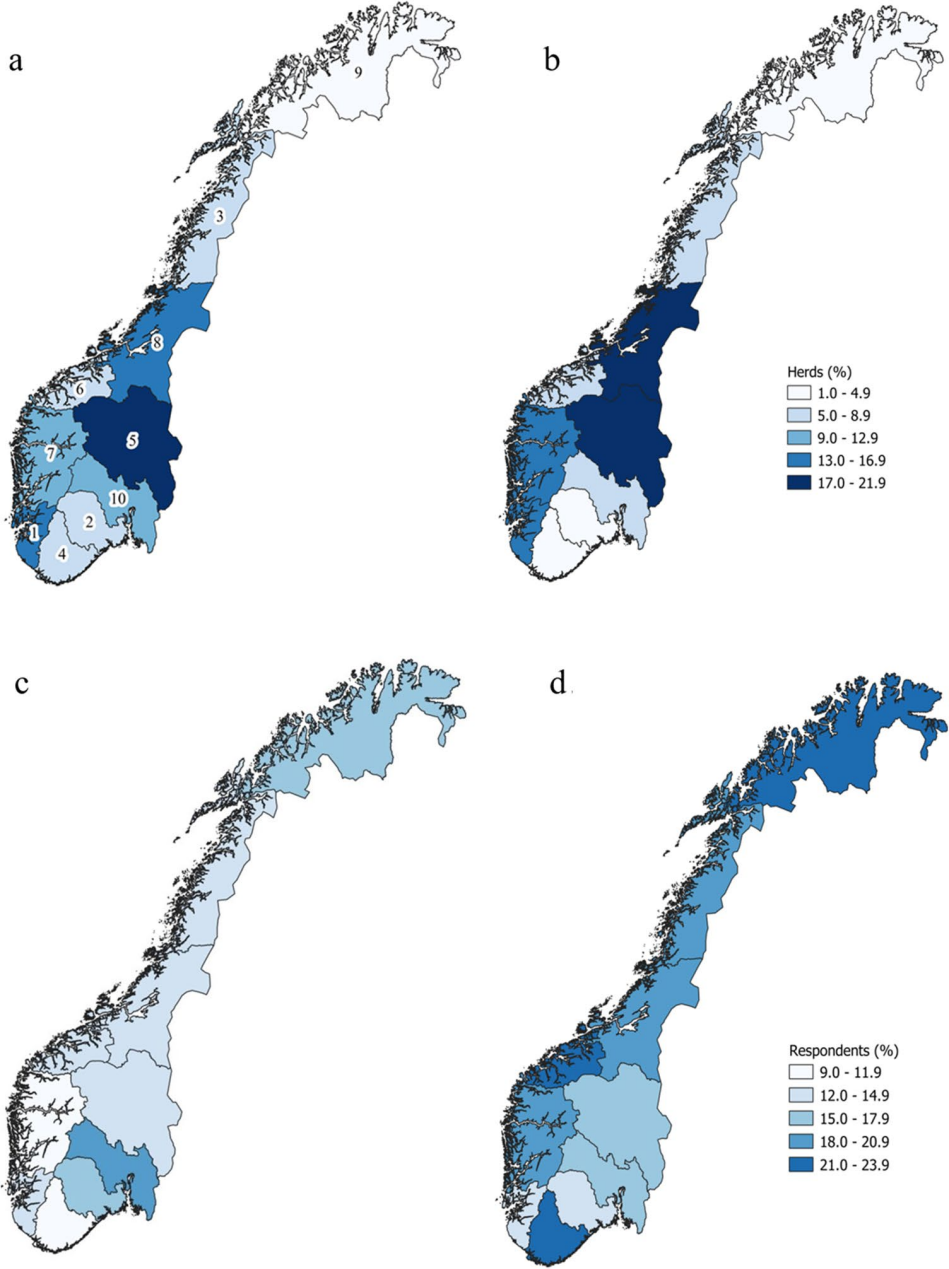
Geographical distribution of cattle herds in Norway and respondents relative to the census population of herds in each county. The geographical distribution of Norwegian cattle herds shown as the relative frequency (%) in each county for **a** Beef cattle farmers and **b** Dairy cattle farmers. Respondents (%) relative to census population of herds in each county for **c** Beef cattle farmers and **d** Dairy cattle farmers. In all maps, Oslo and Viken counties were merged as Oslo only had 2 milk production units in total. 1: Rogaland, 2: Vestfold og Telemark 3: Nordland 4: Agder 5: Innlandet 6: Møre og Romsdal 7: Vestland 8: Trøndelag 9: Troms og Finnmark 10: Oslo and Viken

The number of respondents to the dairy herd questionnaire was 1356 (Table 1), resulting in a response rate of 30.7%. This corresponds to 18% of all dairy herds in Norway in 2019. The respondents represent all 11 counties in Norway, with the majority of respondents coming from the west coast and inland areas (Fig. 1) corresponding with the general herd distribution of DC in Norway. The mean age of the respondents was 49 (range 20–76) years, and their average dairy farming experience was 20 (range 0–40) years.

**Table 1:** General management characteristics of Norwegian dairy and beef cattle herds in present survey

	Dairy cattle	Beef cattle
Number of herds	1356	745
Median herd size in cow years (range)	23 (5–122)	17 (1–163)
Number of farms with organic farming (%)	8 (0.6)	49 (7)
Number of farms with sheep in the farm unit (%)	258 (19)	215 (29)

Range or percentage of respondents are given in brackets

#### Beef cattle respondents

In the beef-herd questionnaire, the number of respondents was 745, resulting in a response rate of 20.5%. This represents 14% of the beef herds in Norway in 2018. The respondents represent all 11 counties in Norway, with most respondents from the inland and central regions (Fig. 1) corresponding with the general herd distribution of BC in Norway.

### Characteristics of the pasture and grazing time

Home pasture is agricultural land that can be used as pasture and is not harvested by the use of machines. It is usually a fenced area close to the farm unit and may be cultivated or uncultivated. Home pasture was used for most of the pasture period for both dairy and beef herds (Table 2), with the widest application for FSG (81% (1097/1348). In BC herds, 66% (437/662) used mainly home pasture. Approximately an equal number of DC respondents stated May or June as month of turnout for FSG turnout, whereas dairy cows and BC were let out in May for the majority of herds.

**Table 2:** Total pasture time, turnout time and the pasture type most frequently used

Mean total pasture time in months	Dairy cattle (n = 1356)				Beef cattle (n = 745)	
	First season grazers		Second season grazers and cows		Mean	SD
	Mean	SD	Mean	SD		
	4.15	1.35	4.31	1.30	5.35	2.36
	n	%	n	%	n	%
Most frequently used pasture						
Home pasture (cultivated)	210	16	269	20	90	14
Home pasture (uncultivated)	878	65	642	48	347	52
Rangeland, only one herd	151	11	236	17	134	20
Rangeland, several herds	109	8	203	15	70	11
Other	N/A	N/A	N/A	N/A	21	3
Total	1348		1350		662	
Turnout time on pasture						
≤ March			2	0.1	1	0.1
April	16	1	29	2	35	5
May	630	47	743	55	382	58
June	654	48	528	39	164	25
July	51	4	40	3	1	0.2
≥ August	2	0.1	3	0.1	0	0
Pastured all year	N/A	N/A	N/A	N/A	78	12
Total	1353		1345		661	

For dairy herds, the frequencies are shown separately for first-season grazers (FSG) and second-season grazers (SSG) or older

*SD* standard deviation; *N/A* not applicable as this option was not present in the questionnaire

### Pasture management

**Fig. 2:**
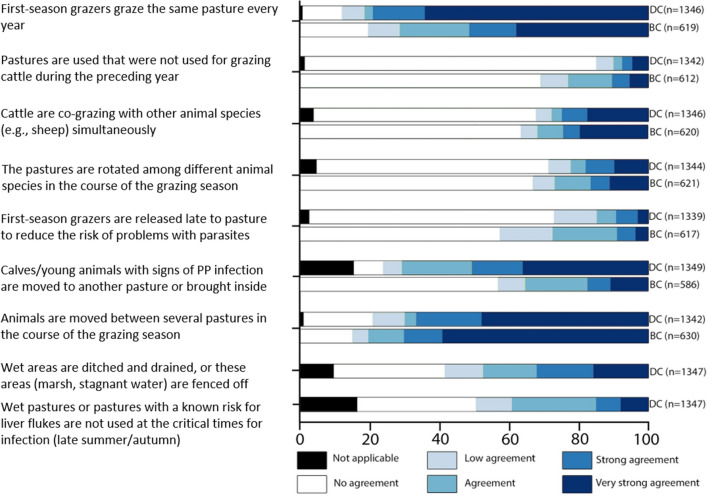
Stack-bar figure with descriptive information about pasture management in Norwegian dairy cattle (DC) and beef cattle (BC). Farmers are stating extent of agreement with the statements in the column on the left concerning their pasture management practices. *PP* pasture parasites

The responses to statements regarding pasture management that can potentially contribute to a high or low parasite burden are summarized in Fig. 2.

#### Use of the same pasture for FSG every year and late turnout of FSG to pasture

BC farmers stated that the FSG are grazed on the same pasture every year with strong or very strong agreement with their practice in 52% (319/619) of the herds, while the corresponding number in DC herds was 79% (1066/1346). Both groups of farmers generally replied that they did not use late turnout to reduce the risk of infection with parasites in FSG. Within the groups of both BC and DC stating a strong to very strong agreement to the practice of late turnout, 30% of BC (30/55) and 24% of DC (29/123) nevertheless reported turnout before June.

#### Co-grazing and rotation of different animal species on pasture

Co-grazing of different animal species on the same pasture at the same time occurred infrequently. Over 68% (922/1346) of DC and 64% (393/620) of BC respondents reported no co-grazing. More frequent co-grazing of different animal species was identified in the herds where sheep were included in the production unit, as 45% (90/196) and 59% (151/257) of BC and DC respondents, respectively, reported that co-grazing strongly or very strongly agreed with their practice. Sequential pasture rotation between different animal species during the grazing season was most common in DC herds with sheep, where 47% (121/256) of respondents noted that this strongly or very strongly described their practice, compared to 11% (118/1075) in herds without sheep. Annual rotation of pastures between different livestock species (e.g., sheep and cattle) was rarely used, with no or low application in 83% (1114/1342) and 85% (522/616) of DC and BC farmers, respectively. This variable also increased when there were sheep in the production unit, as the farmers answering that this measure was applied with strong or very strong agreement doubled from 7% (43/616) to 15% (28/194) in BC herds and from 5% (72/1342) to 15% (37/254) in DC herds.

#### Leaving the pasture unused for grazing cattle for one year and rotation of pasture

The use of pastures that had not been grazed by cattle the preceding year was reported as rare, with 77% (471/612) of BC and 89% (1194/1344) of DC farmers stating no or very low agreement with this measure. However, changing pasture during the grazing season was common, as 67% (896/1342) and 70% (443/630) of DC and BC farmers, respectively, reported a strong or very strong application of this practice.

#### Co-grazing of different age-groups within the herd

Co-grazing of different age-groups on the same grazing area varied considerably between DC and BC herds. Adult BC are often pastured with suckling calves and other young animals, amounting to 79% (526/663) of the BC herds in this survey. In DC herds, however, only 28% of herds reported co-grazing of calves and FSG with SSG and adult animals.

#### Pasture management on wet pastures

Among DC herds, 32% (432/1346) reported that using practical measures, such as ditching or drainage, or fencing off wet pastures or high-risk areas for infection for liver fluke strongly or very strongly described their practice. Limiting the use of these high-risk areas as pastures in the critical times for infection (late summer or autumn) were less frequent; only 15% (202/1347) reported a strong or very strong application of this practice. No regional differences nor an increased applicability among farmers who suspected presence of liver flukes in the herd were found. BC farmers were not asked about measures to limit grazing on wet pastures.

### Perception of problems related to pasture parasites

When DC farmers were asked to specify which parasites cause problems in their herds (Table 3), 4% answered “worms” (nematodes) and 5% responded with “liver flukes”. Approximately 15% of the farmers were unaware whether worms were present in their herds, and a similar proportion was found for awareness of flukes present in the herd. Reports received from abattoirs regarding liver flukes or liver fluke-related damage to the liver were registered by the respondents in 14% of the DC herds. In the BC herds, 17% reported that liver fluke infections occurred or that they had received abattoir reports of liver flukes. The county of Rogaland, a dairy-dense district in the southwest of Norway, had the highest report of both, as 13% (17/135) reported to have worms while 27% (38/139) reported that liver flukes infected their DC herds.

**Table 3:** The farmers’ perceptions of which parasites and clinical signs are present in the herd

Which parasite or clinical sign of parasite infection do you experience in your herd?	Dairy cattle farmers					
	Yes		No		Unknown	
	n	%	n	%	n	%
Worms n = 1324	58	4	1062	80	202	15
Liverflukes n = 1328	65	5	1041	78	222	17
Abbatoir reports of liver flukes n = 1332	191	14	1096	82	34	3
Diarrhea on pasture n = 1329	82	6	1188	89	59	4
Decreased average daily gain on pasture n = 1333	150	11	1072	80	111	8

	Beef cattle farmers (n = 539)					
	Yes		No		Unknown	
	n	%	n	%	n	%
Liver flukes or abbatoir reports of liver flukes	94	17	445	83	N/A	N/A
Diarrhea on pasture	20	4	519	96	N/A	N/A
Decreased average daily gain on pasture	24	4	515	96	N/A	N/A

*N/A* not applicable as this option was not present in the questionnaire

The overall perception of problems related to PP was another question directed only to DC farmers, where 60% (812/1356) of farmers stated that they did not have a problem with PP in their herd, and only 1% (17/1356) reported having a serious or very serious problem with PP. Among the proportion of respondents reporting a problem, 11% (55/522) specified that their cattle had worms (nematodes) while 12% (62/529) had flukes and 28% (145/530) had abattoir reports of liver fluke-related damage. The proportion of these farmers that were unaware of the PP status of their herds, amounted to 28% for both worms (148/522) and liver flukes (148/529). No marked differences in the pasture-management regime were observed between DC farmers claiming to have a problem with PP and farmers indicating that there was no problem with PP in their herds.

### Treatment regime

The proportions of farmers who reported using anthelmintics against worms in their herd in 2019 (DC) or 2017 (BC) were 53% of DC farmers (Table 4) and 34% (190/562) of BC farmers. This question did not differentiate between strategic or therapeutic treatment. In both DC and BC herds, 11% of farmers treated for flukes (55/505 of BC farmers). Specifications regarding the age group receiving anthelmintic treatment were obtained for DC farmers and indicate that treatment was targeted to FSG.

**Table 4:** Treatment with anthelmintics against gastrointestinal nematodes (GIN) and liver flukes in dairy cattle herds

	Dairy cattle farmers	
	n	%
Frequency of farmers who treat against GIN (n = 1344)	711	53
Age categories receiving treatment against GIN		
First-season grazers (n = 705)	673	95
Second-season grazers (n = 633)	127	20
Cows (n = 615)	23	4
Frequency of farmers who treat against liver flukes (n = 1345)	147	11
Age categories receiving treatment against liver flukes		
First-season grazers (n = 147)	125	85
Second-season grazers (n = 129)	36	28
Cows (n = 127)	7	6

Should clinical signs of PP infection during the grazing season be observed in a cattle herd (Table 5), prophylactic treatment with a bolus in the upcoming grazing season was a frequent choice in DC herds, as more than half of the farmers chose this application. In BC herds, treatment of only symptomatic animals was done in 45% of the herds. The concurrent use of a diagnostic faecal sample to establish the cause was only reported by 5% and 16% of DC and BC farmers, respectively. In fact, only 9% of BC farmers reported ever having collected a diagnostic sample for PP, and, among those, 48% (26/55) noted that the reason for the test was to assess the need for treatment in the herd.

**Table 5:** Routines regarding treatment for pasture parasites if symptoms of parasitic infection occur during the grazing season

Treatment routines	Dairy cattle farmers, n = 354		Beef cattle farmers n = 611	
	n	%	n	%
Treatment only of symptomatic animals	72	20	272	45
Treatment of symptomatic animals and all in that age group	70	20	72	12
Treatment of all individuals	54	15	79	13
Prophylactic treatment of the exposed age group next season	198	56	N/A	N/A
Diagnostic faecal sample is taken	16	5	97	16
No particular measures	54	15	141	23

*N/A* not applicable as this option was not present in the questionnaire

In the group of DC farmers where PP were not perceived as a problem, 44% (356/806) dewormed their herd and 8% (64/806) gave flukicides. The proportion of farmers using treatment was higher in herds where PP was perceived as a problem than among those who did not perceive PP as a problem. Among DC farmers who considered PP to be a problem, 66% (356/538) used dewormers, and 16% (83/529) reported administering flukicides. In Rogaland, 65% (93/144) of DC farmer respondents reported having a problem with PP. In this area, use of anthelmintics was the highest, with 72% (103/143) of DC farmer respondents treating their cattle for worms.

### Sources of information and advice regarding parasites for Norwegian farmers

**Fig. 3:**
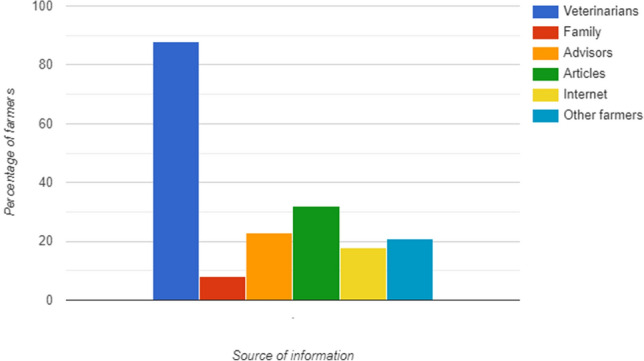
Preference of source of information regarding pasture parasite management and treatment for 643 beef cattle farmers and 1339 dairy cattle farmers. Multiple answers possible

Veterinarians were reported as being central in supply of information and guidance for farmers regarding parasite management in the cattle herd, as seen in Fig. 3. Other sources of information were reported as being used to a lesser extent. In the group of DC farmers where treatment against nematodes was reported, the proportion of farmers who were encouraged to perform diagnostic tests before routine treatment was 3% (23//706), while those who were encouraged based on suspicion of parasites was 11% (80/706). Of all DC farmers who treated their herd against nematodes, 84% (590/706) reported never to be encouraged by neither veterinarian nor advisors to perform diagnostic faecal sample for parasites.

## Discussion

This study identified several management factors in Norwegian cattle herds that can influence the parasite pressure related to PP. This included frequent use of home pasture, mainly for FSG. Furthermore, grazing cattle of all ages were usually released onto pastures that had also been stocked during the previous year, and the time of turnout may expose these animals to overwintering larvae on pasture. The overall perception of problems caused by PP was considered minor by DC farmers. However, 52% of DC farmers and 34% of BC farmers reported use of anthelmintic treatments against worms and 11% of the respondents treated for flukes. The treatment regime rarely involved diagnostic tests.

The turnout of FSG onto the same pasture every year is an important risk factor for infection with overwintering parasites, as naïve animals are more likely to be infected when grazing highly stocked, contaminated pastures [28, 29]. Furthermore, the density of animals on pasture varies according to the use of home pasture or rangeland pasture. On rangeland pasture, the density of animals is low, and thus the build-up of a large PP infection burden in a smaller area is avoided. This is in contrast with home pasture, where the density of animals is likely to be higher, which could be a contributing factor towards greater exposure to parasite transmission stages. A study investigating heifers’ individual milk samples for antibodies against *O. ostertagi* found ODR-values to be significantly increased with high stocking density at first grazing [8].

In temperate countries where animals are housed during the winter, such as Norway, the date of turnout influences exposure to overwintering parasites on pasture. If livestock are held inside until nematode larvae on the pasture have succumbed, the subsequent contamination level will be minimal [28]. In Norway, the number of larvae on pastures stocked the year before gradually decreases during the spring, until they have all died by the end of June [30]. Our survey detected a turnout centred on May and June in both DC and BC herds, and late release to pasture to reduce the risk of exposure to parasites was rarely used. Management factors related to availability and cost of food as well as welfare regulations may limit the usefulness of this strategy. However, in combination with frequent release of FSG onto the same pasture every year, early release may provide grazing conditions with a risk of high exposure of FSG to overwintering larvae soon after turnout.

Pastures that have not been grazed by cattle for at least a year previously can be used with little risk of exposure to high infection pressure. The restricted availability of pasture for many Norwegian cattle production units probably prevents the wide application of this strategy. Cattle movement to different pastures is used by most farmers to provide animals with better quality grazing throughout the season. As the build-up of infection occurs progressively on pasture, this measure also results in animals moving from highly contaminated to minimally contaminated pasture during the grazing season, thereby reducing infection pressure.

Pasture management involving mixed grazing of different species of livestock, either simultaneously or by rotation, is an important preventive strategy where significant benefits have been achieved in GIN control for both sheep and cattle. This strategy draws on exploitation of host specificity, where parasite species that are pathogenic in one host species are less pathogenic and prolific, or do not infect other species [29]. One study demonstrated that antibodies against *O. ostertagi* in milk samples from heifers significantly decreased when heifers had co-grazed with sheep [8]. According to our findings, such regimes are not widely used in Norway, even in holdings with sheep present. Practical concerns regarding grazing management of the two species probably limits wider application of this strategy. An important note is that this practice will not benefit, and may even intensify, the risk of infection with liver flukes, as sheep are good hosts for *F. hepatica*.

As adult animals have lower egg shedding due to acquired immunity against GIN, co-grazing FSG with SSG and adult animals is one strategy to reduce infection pressure on pasture [29]. However, we found it to be rarely practiced in Norwegian DC herds. Grazing management factors related to pasturing animals in lactation and impracticalities related to co-grazing animals of different age categories are probable reasons for the restricted use.

The major control measures against fasciolosis consist of anthelmintic administration and environmental measures, such as drainage and fencing-off wet pasture areas [31]. These pasture-management measures are supported by studies concluding that poorly drained soil types are associated with high risk of exposure to liver fluke metacercaria [10, 32, 33]. Limitation or strategic timing of grazing wet pastures can also reduce the risk of a high infection pressure. Farmers who reported fluke infection or treatment for flukes showed regional differences, coinciding with knowledge about the spatial distribution of liver flukes in Norway. However, the use of such pasture measures in these regions was not reflected in the questionnaire responses.

The mean duration of the grazing season for Norwegian cattle was relatively short compared with those of other European countries [34]. A survey investigating the prevalence of *O. ostertagi* in Europe using BTM samples found it to be significantly higher in central European countries than in Sweden [7], which has a similar climate to Norway. In addition, Sweden had the shortest mean grazing season in the study with 4.5 months; this is comparable to Norwegian practices according to the findings in our study. The period on pasture will be a relevant factor for influencing the impact of parasitism in a future with climate changes. Warmer autumn and spring seasons, resulting in a prolonged grazing season, may increase the duration of exposure to helminth parasites. Greater exposure to *F. hepatica* metacercaria in late autumn and earlier exposure to GIN larvae in the spring could thus be a consequence of climate change [35]. Studies have detected that increased exposure to pasture was associated with higher antibody levels against *O. ostertagi* [6, 7, 9] and, in Sweden, duration of grazing period was a significant predictor of exposure to *F. hepatica* [36]. However, another study from Ireland [31] did not detect a significance association between length of the pasture season and *F. hepatica* exposure.

The overall perception of problems related to PP among DC farmers was low, and, in general, PP appeared to be considered a minor problem by these farmers. Although 40% of DC farmers stated that PP presented a problem to some extent in their herds, only a few respondents stated specifically whether their herds had a problem due to infection by GIN or liver flukes, and many were unaware of the parasite situation in their herd. This can be related to the low use of diagnostic faecal samples to ascertain the level of parasite infection in Norwegian cattle production systems prior to implementing a treatment regime. This finding is supported by Norwegian laboratories receiving faecal samples for diagnostic purposes (personal communication: Inger Sofie Hamnes, Norwegian Veterinary Institute; Parasitology Laboratory, NMBU).

Although most DC farmers consider their problems with PP to be minor, a large proportion use anthelmintics against nematodes. The use of anthelmintic treatment increased according to the farmers’ perception of whether they experienced problems related to PP. However, it was not within the scope of this study to assess the causal relationship between these two factors. Whether farmers treat their animals because they have problems or because they want to avoid problems warrants further investigation. However, it should be noted that a larger proportion answered positively to using prophylactic treatment in the upcoming season on suspicion of PP infestation.

The regional differences regarding perception of problems with PP and treatment frequency reflect previous knowledge about occurrence of PP in Norway [4, 30]. The Norwegian coastal area, which is relatively warm and damp, is a more favourable environment for parasites’ larval development and survival, and also provides a longer pasture season than in northern areas or high-altitude regions. The high rainfall and marshy pastures are associated with increased risk of exposure of *F. hepatica* [33]. Country-level similarities in the distribution and occurrence of *O. ostertagi* and *F. hepatica* might suggest that the two helminth infections share common dependencies on pasture management as well as on climatic conditions [7], which is mirrored in the answers from the dairy farmers in our study.

Our study revealed a high level of regard for the expertise and advice provided by veterinarians in the farmers’ decision-making processes. This may reflect the relatively strict regulations regarding use of anthelmintics in Norway, requiring a veterinary prescription, enabling veterinarians to influence helminth control in any given farm. The role of the veterinarian agrees with results from studies in other countries such as Sweden [37] and the UK [20]. Alternative sources of information listed in our questionnaire appear to be less used and are probably supplementary to veterinary advice. However, the vast proportion of DC farmers treating their herd with anthelmintics against nematodes were doing so without being encouraged to perform faecal diagnostic samples. This suggests that the guidance provided may not be optimal and may even indicate a lack of knowledge among veterinarians and other advisory personnel available to the farmers.

## Limitations and validation of the study

The response rate of this survey was somewhat limited, potentially introducing a non-response bias. There could be a tendency of more engaged farmers to participate in the survey. As the subject was clearly described as being focused on PP, it is likely that the farmers interested in this subject were more motivated to take part than farmers considering this issue as less important in their farm management. The median herd sizes of responding DC and BC farmers were comparable to the median herd sizes of the background populations. The proportion of organic farming units showed a weak deviation from the country means in DC (3.7% in 2020 [38]) and BC (4.7% in 2018 [39]). However, the survey population of responding farmers was widespread and showed a strong correlation between number of respondents and geographical distribution of both dairy and beef cattle farmers in Norway. The median age of a dairy farmer included in this survey was 49 years, corresponding closely with the median age of a Norwegian farmer being 52 years in 2018 [40].

The self-reported information regarding pasture use and treatment may be influenced by social desirability, as well as subject to recall bias resulting in an inaccurate response. However, as pasture management is often performed in a similar way every year, we consider this information to be reliable. A further limitation of a closed-ended questionnaire is the restriction on obtaining nuanced insights into the complex systems of management and grazing and limited representation of management is to be expected. The study aimed to describe the farmers’ perception of problems related to PP and the associated management employed in the production unit. The interplay of perception of problems and the use of anthelmintics was therefore difficult to investigate by a web-based survey, and a set of complementary in-depth personal interviews may provide more useful clues to address this question.

## Conclusions

Known risk factors for PP exposure, e.g., use of the same pasture every year for FSG and frequent use of home pasture were identified. The majority of farmers perceive the problems caused by PP to be minor or not occurring in their herd. However, the study indicated a general lack of knowledge and awareness about the status of PP among Norwegian farmers, which is supported by the infrequent use of faecal samples for diagnostic or surveillance purposes. Prophylactic treatment with anthelmintics seems to be a common practice among Norwegian dairy farmers.

## Supplementary Information

**Additional file 1: **Questionnaire to dairy cattle farmers.

**Additional file 2: **Questionnaire to beef cattle farmers.

## Data Availability

The datasets generated and analysed during the current study are not publicly available due to GDPR reasons, but are available from the corresponding author on reasonable request.

## Abbreviations

AR: Anthelmintic resistance
BC: Beef cattle
BTM: Bulk tank milk
DC: Dairy cattle
FSG: First season grazers
GIN: Gastrointestinal nematodes
ODR: Optical density ratio
PP: Pasture parasites

## References

[1] Morgan ER, Charlier J, Hendrickx G, Biggeri A, Catalan D, Von Samson-Himmelstjerna G (2013). Global change and helminth infections in grazing ruminants in Europe: impacts, trends and sustainable solutions. Agriculture.

[2] Forbes AB, Vercruysse J, Charlier J (2008). A survey of the exposure to *Ostertagia ostertagi* in dairy cow herds in Europe through the measurement of antibodies in milk samples from the bulk tank. Vet Parasitol.

[3] Ducheyne E, Charlier J, Vercruysse J, Rinaldi L, Biggeri A, Demeler J (2015). Modelling the spatial distribution of *Fasciola hepatica* in dairy cattle in Europe. Geospat health.

[4] Tharaldsen J, Helle O (1984). Epidemiological investigations of trichostrongylid infections in young cattle in different parts of Norway. Acta Vet Scand.

[5] Tharaldsen J (1976). The epidemiology of trichostrongylid infections in young cattle in Norway. Acta Vet Scand.

[6] Merlin A, Chauvin A, Madouasse A, Froger S, Bareille N, Chartier C (2016). Explaining variability in first grazing season heifer growth combining individually measured parasitological and clinical indicators with exposure to gastrointestinal nematode infection based on grazing management practice. Vet Parasitol.

[7] Bennema SC, Vercruysse J, Morgan E, Stafford K, Höglund J, Demeler J (2010). Epidemiology and risk factors for exposure to gastrointestinal nematodes in dairy herds in northwestern Europe. Vet Parasitol.

[8] Bellet C, Green MJ, Bradley AJ, Kaler J (2018). A longitudinal study of gastrointestinal parasites in English dairy farms. Practices and factors associated with first lactation heifer exposure to *Ostertagia**ostertagi* on pasture. J Dairy Sci.

[9] Charlier J, Claerebout E, Mûelenaere ED, Vercruysse J (2005). Associations between dairy herd management factors and bulk tank milk antibody levels against *Ostertagia ostertagi*. Vet Parasitol.

[10] Bennema SC, Ducheyne E, Vercruysse J, Claerebout E, Hendrickx G, Charlier J (2011). Relative importance of management, meteorological and environmental factors in the spatial distribution of *Fasciola hepatica* in dairy cattle in a temperate climate zone. Int J Parasitol.

[11] Charlier J, van der Voort M, Kenyon F, Skuce P, Vercruysse J (2014). Chasing helminths and their economic impact on farmed ruminants. Trends Parasitol.

[12] Vercruysse J, Claerebout E (2001). Treatment vs non-treatment of helminth infections in cattle: defining the threshold. Vet Parasitol.

[13] Svensson C, Hessle A, Höglund J (2000). Parasite control methods in organic and conventional dairy herds in Sweden. Livest Prod Sci.

[14] Gasbarre LC (2014). Anthelmintic resistance in cattle nematodes in the US. Vet Parasitol.

[15] Rose H, Rinaldi L, Bosco A, Mavrot F, de Waal T, Skuce P (2015). Widespread anthelmintic resistance in European farmed ruminants: a systematic review. Vet Rec.

[16] Sutherland IA, Leathwick DM (2011). Anthelmintic resistance in nematode parasites of cattle: a global issue?. Trends Parasitol.

[17] Charlier J, Morgan ER, Rinaldi L, van Dijk J, Demeler J, Höglund J (2014). Practices to optimise gastrointestinal nematode control on sheep, goat and cattle farms in Europe using targeted (selective) treatments. Vet Rec.

[18] Vercruysse J, Charlier J, Van Dijk J, Morgan ER, Geary T, von Samson-Himmelstjerna G (2018). Control of helminth ruminant infections by 2030. Parasitology.

[19] Domke AV, Chartier C, Gjerde B, Höglund J, Leine N, Vatn S (2012). Prevalence of anthelmintic resistance in gastrointestinal nematodes of sheep and goats in Norway. Parasitol Res.

[20] Garforth C (2015). Livestock keepers’ reasons for doing and not doing things which governments, vets and scientists would like them to do. Zoonoses Pub Health.

[21] Vande Velde F, Claerebout E, Cauberghe V, Hudders L, Van Loo H, Vercruysse J (2015). Diagnosis before treatment: identifying dairy farmers’ determinants for the adoption of sustainable practices in gastrointestinal nematode control. Vet Parasitol.

[22] Domke AV, Chartier C, Gjerde B, Leine N, Vatn S, Osterås O (2011). Worm control practice against gastro-intestinal parasites in Norwegian sheep and goat flocks. Acta Vet Scand.

[23] Gravdal M, Robertson LJ, Tysnes KR, Höglund J, Chartier C, Stuen SJP (2021). Treatment against helminths in Norwegian sheep: a questionnaire-based survey. Parasite.

[24] Landbruksdirektoratet. PT-900 Antallsstatistikk. 2020. https://www.landbruksdirektoratet.no/filserver/statistikkgrafikk/pt-900_del1_2019_land.html. Accessed 10 Aug 2020.

[25] Espetvedt MN, Reksen O, Rintakoski S, Østerås O (2013). Data quality in the Norwegian dairy herd recording system: agreement between the national database and disease recording on farm. J Dairy Sci.

[26] European Parliament and Council of European Union (2016) Regulation (EU) 2016/679. https://eur-lex.europa.eu/legal-content/EN/TXT/HTML/?uri=CELEX:32016R0679&from=EN. Accessed 01 Oct 2020.

[27] QGIS Geographic Information System. Open Source Geospatial Foundation. https://www.qgis.org/en/site/. Accessed 15 Jan 2021.

[28] Armour J (1980). The epidemiology of helminth disease in farm animals. Vet Parasitol.

[29] Waller PJ (2006). Sustainable nematode parasite control strategies for ruminant livestock by grazing management and biological control. Anim Feed Sci Technol.

[30] Parasittar hos storfe (Cattle Parasites, compendium). 2011. http://bk.gjerde.name/index.php/component/phocadownload/category/14-kompendium?download=117:storfepar-2011. Accessed 10 Jan 2021.

[31] Selemetas N, Phelan P, O’Kiely P, de Waal T (2015). The effects of farm management practices on liver fluke prevalence and the current internal parasite control measures employed on Irish dairy farms. Vet Parasitol.

[32] Selemetas N, Phelan P, O’Kiely P, de Waal T (2014). Weather and soil type affect incidence of Fasciolosis in dairy cow herds. Vet Rec.

[33] Howell A, Baylis M, Smith R, Pinchbeck G, Williams D (2015). Epidemiology and impact of *Fasciola hepatica* exposure in high-yielding dairy herds. Prev Vet Med.

[34] Phelan P, Morgan E, Vineer H, Grant J, O'Kiely P (2015). Predictions of future grazing season length for European dairy, beef and sheep farms based on regression with bioclimatic variables. J Agr Sci.

[35] van Dijk J, Sargison ND, Kenyon F, Skuce PJ (2010). Climate change and infectious disease: helminthological challenges to farmed ruminants in temperate regions. Animal.

[36] Novobilský A, Sollenberg S, Höglund J (2015). Distribution of *Fasciola hepatica* in Swedish dairy cattle and associations with pasture management factors. Geospat Health.

[37] Fischer K, Sjöström K, Stiernström A, Emanuelson U (2019). Dairy farmers’ perspectives on antibiotic use: a qualitative study. J Dairy Sci.

[38] Kjøttets tilstand. 2020. https://www.animalia.no/contentassets/8516b3a48201409297db211f33bf6c76/kt20-komplett-origi-web.pdf. Accessed 15 Aug 2021.

[39] Kjøttets tilstand. 2018. https://www.animalia.no/contentassets/ea773e1d55294823802670cac747a264/kjottets-tilstand-2018.pdf. Accessed 15 Aug 2021.

[40] Statistics Norway. Statistikkbanken. https://www.ssb.no/statbank/table/05976/tableViewLayout1/. Accessed 15 Aug 2020.

