# Supplementary material for: Gastrointestinal nematodes and Fasciola hepatica in Norwegian cattle herds: a questionnaire to investigate farmers’ perceptions and control strategies
Source: Acta Vet Scand. 2021 Dec 4;63:52. doi: 10.1186/s13028-021-00618-7 (PMC8645080; doi:10.1186/s13028-021-00618-7)
Supplement: Supplementary file 1 — Additional file 1: Questionnaire to dairy cattle farmers. [file 13028_2021_618_MOESM1_ESM.docx]

QUESTIONNAIRE FOR DAIRY CATTLE FARMERS

January 2020

__________________________________________________________________________________

**1. Production number (10 digits)­­­­­­­­­­­­­­­­**

­__________________________________

**2. Are there other animals on your farm in addition to dairy cattle? Multiple answers possible**

- Beef Cattle
- Goat
- Sheep
- Horses
- Pigs
- Llamas
- Alpacas
- Other ______________________

**3. Is your production unit organic?**

- Yes
- No

**4. Are calves and young animals (defined as animals <24 months in their first pasture season) usually co-pastured with cows? Only one answer**

- Yes
- No

**5. What type of pasture is used for first-season grazers? If several types are used, choose the one used for the majority of the pasture period and most of the animals in 2019.**

- Home pasture, cultivated
- Home pasture, not cultivated or harvested
- Rangeland – only together with animals from the same herd
- Rangeland – together with animals from other herds
- Other ______________________________

**6. What type of pasture is used for second-season grazers and cows? If several types are used, choose the one used for the majority of the pasture period and most of the animals in 2019.**

- Home pasture, cultivated
- Home pasture, not cultivated or harvested
- Rangeland – only together with animals from the same herd
- Rangeland - together with animals from other herds
- Other ______________________________

**7. When are calves/first-season grazers usually turned out onto pasture?**

Drop-down menu: January – December

**8. For how many months are calves/first-season grazers usually on pasture?**

Drop-down menu: 1-12 months

**9. When are calves/first-season grazers usually brought inside from the pasture?**

Drop-down menu: January – December

**10. When are second-season grazers and cows usually turned out onto pasture?**

Drop-down menu: January – December

**11. For how many months are second-season grazers and cows usually on pasture?**

Drop-down menu: 1-12 months

**12. When are second-season grazers and cows usually brought inside from the pasture?**

Drop-down menu: January – December

**13. How well do the following sentences describe pasture use for your herd? On a scale of 1 to 5, where 1 = no agreement and 5= total agreement, or N/A = not applicable, cross off the most appropriate box – give only one answer per question.**

|  | | 1 | 2 | | 3 | | 4 | | 5 | | N/A | |  |
| --- | --- | --- | --- | --- | --- | --- | --- | --- | --- | --- | --- | --- | --- |
| Pastures are used that were not used for grazing cattle during the preceding year | | ❑ | ❑ | | ❑ | | ❑ | | ❑ | | ❑ | |  |
| Animals are moved between several pastures in the course of the pasture season | | ❑ | ❑ | | ❑ | | ❑ | | ❑ | | ❑ | |  |
| First-season grazing calves/young animals graze the same pasture every year | | ❑ | ❑ | | ❑ | | ❑ | | ❑ | | ❑ | |  |
| First-season grazing calves/young animals are released late to pasture to reduce the risk of problems with parasites | | ❑ | ❑ | | ❑ | | ❑ | | ❑ | | ❑ | |  |
| In the course of the pasture season, cattle and other animal species (e.g., horses, small ruminants) co-graze on the same pasture simultaneously | | ❑ | ❑ | | ❑ | | ❑ | | ❑ | | ❑ | |  |
| In the course of the pasture season, cattle and other animal species (e.g., horses, small ruminants) co-graze on the same pasture, but not simultaneously | | ❑ | ❑ | | ❑ | | ❑ | | ❑ | | ❑ | |  |
| Each year, the animal species (e.g., cattle, sheep, horses etc) grazing a particular pasture is changed | | ❑ | ❑ | | ❑ | | ❑ | | ❑ | | ❑ | |  |
| Wet areas are ditched and drained, or these areas (marsh, stagnant water) are fenced off | | ❑ | ❑ | | ❑ | | ❑ | | ❑ | | ❑ | |  |
| Wet pastures or pastures with a known risk for liver flukes are not used at the critical times for infection (late summer/autumn) | | ❑ | ❑ | | ❑ | | ❑ | | ❑ | | ❑ | |  |
| Feeding areas are regularly moved around the pasture | ❑ | | | ❑ | | ❑ | | ❑ | | ❑ | | ❑ | |
| Calves/young animals with signs of pasture parasites infection (e.g., diarrhoea) are moved to another pasture or brought inside | | ❑ | ❑ | | ❑ | | ❑ | | ❑ | | ❑ | |  |

**14. Do you consider pasture parasites to be a problem in your herd? Only one answer**

- Not a problem
- A minor problem
- A problem
- A serious problem
- A very serious problem

**15. Are any of these parasites or symptoms a problem in your herd?**

Yes No Unknown

| Intestinal worms (roundworm) | ❑ | ❑ | ❑ |
| --- | --- | --- | --- |
| Liver flukes (liver fluke disease that occurs in mid-winter/spring with reduced weight and potentially reduced milk yield) | ❑ | ❑ | ❑ |
| Abattoir reports of liver-fluke findings | ❑ | ❑ | ❑ |
| Diarrhoea in calves/young animals on pasture | ❑ | ❑ | ❑ |
| Poor growth in calves/young animals on pasture | ❑ | ❑ | ❑ |

For answers of “yes” to any of the alternatives in 15A, the respondent is routed to 15B

**15B. What do you do if symptoms that could be caused by pasture parasites occur in the grazing period? The term “Treat” here refers to administration of anthelmintics. Multiple answers possible**

- Treat animals that have symptoms
- Treat animals with symptoms and other animals in the same age-group as those with showing symptoms
- Treat every animal on pasture (every age-group)
- Take a faecal sample to find out whether pasture parasites are causing the symptoms
- Give a preventative bolus to the exposed age-group at next pasture season
- Move the animals to another pasture
- No particular measures
- Other measures: _____________________________________________________________

___________________________________________________________________________

**16. Are you encouraged by your vet or advisor to take faecal samples for investigation for parasites? Multiple answers possible**

- Yes, I am encouraged by my vet prior to routine treatment
- Yes, I am encouraged by my vet on suspicion of parasites
- Yes, I am encouraged by my advisor prior to routine treatment
- Yes, I am encouraged by my advisor on suspicion of parasites
- No, I am not encouraged

**17. Do you treat newly purchased animals with anthelmintics?**

- Yes, always treated
- Yes, sometimes treated
- No
- The animals are already treated when I buy them
- Do not purchase animals from other herds

**18A. Have you used anthelmintics against intestinal worms during the last 12 months on animals that were NOT newly bought in?**

- Yes
- No

If “Yes”, the respondent is routed to question 18B

**18B How many times were the following animal groups treated with anthelmintics in 2019? (Give only one answer per category)**

|  | 1 | 2 | 3 | 4 | 5 | >5 |
| --- | --- | --- | --- | --- | --- | --- |
| Calves/young animals in the first grazing season | ❑ | ❑ | ❑ | ❑ | ❑ | ❑ |

| Young animals (up to 24 months) in the second grazing season | ❑ | ❑ | ❑ | ❑ | ❑ | ❑ |
| --- | --- | --- | --- | --- | --- | --- |
| Cows | ❑ | ❑ | ❑ | ❑ | ❑ | ❑ |

**19A. Have you used anthelmintics against liver flukes during the last 12 months on animals that were NOT newly bought in?**

- Yes
- No

If “Yes”, the respondent is routed to question 18B

**19B How many times were the following animal groups treated with anthelmintics against liver flukes in 2019? (Give only one answer per category)**

|  | 1 | 2 | 3 | 4 | 5 | >5 |
| --- | --- | --- | --- | --- | --- | --- |
| Calves/young animals in the first grazing season | ❑ | ❑ | ❑ | ❑ | ❑ | ❑ |
| Young animals (up to 24 months) in the second grazing season | ❑ | ❑ | ❑ | ❑ | ❑ | ❑ |
| Cows | ❑ | ❑ | ❑ | ❑ | ❑ | ❑ |

**20. Do you weigh animals before treating with anthelmintics against internal parasites? Only one answer**

- Yes, I weigh every animal that is treated
- Yes, I weigh one/few randomly selected
- Yes, I weigh the largest/one of the largest
- Yes, I weigh one middle-sized animal
- Yes, I weigh one of the smallest
- No

**21. What type of anthelmintic/anthelmintic treatment did you use against internal parasites in your herd in 2019? Multiple answers possible**

- Pour-on (the treatment is applied in a stripe along the back of the animal)
- Oral treatment (liquid, paste, powder etc)
- Injection, given by the veterinarian
- Bolus/capsule that is put into the rumen of the animal before it is released onto pasture
- None
- Other _________________________________________________________________________

**22A. Have you noticed that the effect of treatment against internal parasites has been poorer than expected? Only one answer**

- Yes, often
- Yes, sometimes
- Yes, but rarely
- No

In case of any Yes-alternatives, the respondent is routed to 22B

**22B What do you think can be the reason for a poor effect of treatment? Multiple answers possible**

- Incorrect application of substance
- Wrong dosage for weight
- The parasites survive the anthelmintic
- Don’t know

**23. Where do you obtain information/advice about measures and treatment against parasites in your cattle herd? Several answers possible**

- Articles in magazines for farmers
- Internet
- Other farmers
- Veterinarians
- Advisors (for instance, advisors in TINE or abattoirs, Norwegian Agricultural Extension Service, other organizations)
- Family
- Others:_____________________________________________________________________

**24. How old are you?**

Drop down menu

**25. How long (in years) have you worked in dairy cattle production?**

Drop down menu

**26. What is your highest level of education?**

- Primary school
- Upper secondary school – not agricultural studies
- Upper secondary school – agricultural studies
- University

**27. Comments on the questionnaire**

________________________________________________________________________________________________________________________________________________________________________________________________________________________________________________________________________________________________________________________________________
