# Supplementary material for: Gastrointestinal nematodes and Fasciola hepatica in Norwegian cattle herds: a questionnaire to investigate farmers’ perceptions and control strategies
Source: Acta Vet Scand. 2021 Dec 4;63:52. doi: 10.1186/s13028-021-00618-7 (PMC8645080; doi:10.1186/s13028-021-00618-7)
Supplement: Supplementary file 2 — Additional file 2: Questionnaire to beef cattle farmers. [file 13028_2021_618_MOESM2_ESM.docx]

QUESTIONNAIRE FOR BEEF-CATTLE FARMERS

Summer 2018

(Those questions relating to external parasites are not included in this translation)

Are there other animals on your farm in addition to beef cattle?

- Dairy cows
- Goats
- Sheep
- Horses
- Pigs
- Llamas
- Alpacas
- Other ______________________

Is your production unit organic?

- Yes
- No

Were you visited by a veterinarian during 2017?

- Yes
- No

Which of these parasites/symptoms do you consider as a problem in your herd? Multiple answers possible

- Lice
- Tail mange
- Ticks
- Flies, midges, other insects on pasture
- Flies etc inside
- Babesia (destroys red blood cells and causes the urine to be red)
- Liver flukes (have you received report from the abattoir about liver flukes?)
- Lung worm
- Diarrhoea in calves/young animals on pasture
- Diarrhoea in calves/young animals housed inside
- Poor growth in calves/young animals on pasture
- Poor growth in calves/young animals housed inside
- Other_______________________

Do you treat newly purchased animals with anthelmintics?

- Yes, always treated
- Yes, sometimes treated
- No
- The animals are already treated when I buy them
- Do not purchase animals from other herds

Did you treat your herd against internal parasites during 2017 (except in association with purchase of animals). Only one answer

Yes No Don’t know

| Coccidiosis | ❑ | ❑ | ❑ |
| --- | --- | --- | --- |
| Cryptosporidiosis | ❑ | ❑ | ❑ |
| Worms (intestinal worms, pasture parasites) | ❑ | ❑ | ❑ |
| Liver flukes | ❑ | ❑ | ❑ |
| Lungworm | ❑ | ❑ | ❑ |

Other ________________________________________________________________________

How many times were the following animal groups treated against internal parasites during 2017? Only one answer per category

Cows

| 1 | 2 | 3 | 4 | 5 | >5 |
| --- | --- | --- | --- | --- | --- |
| ❑ | ❑ | ❑ | ❑ | ❑ | ❑ |

Calves/young animals in the first grazing season

| 1 | 2 | 3 | 4 | 5 | >5 |
| --- | --- | --- | --- | --- | --- |
| ❑ | ❑ | ❑ | ❑ | ❑ | ❑ |

Young animals in the second grazing season

| 1 | 2 | 3 | 4 | 5 | >5 |
| --- | --- | --- | --- | --- | --- |
| ❑ | ❑ | ❑ | ❑ | ❑ | ❑ |

What type of anthelmintic/anthelmintic treatment did you use in your herd in 2017? Multiple answers possible

- Pour-on (the treatment is applied in a stripe along the back of the animal)
- Oral treatment (liquid, paste, powder etc)
- Injection, given by the veterinarian
- Bolus/capsule that is put into the rumen of the animal before it is released onto pasture
- None
- Other _____________________

Do you weigh your animals before treatment with anthelmintics against internal parasites? Only one answer

- Yes, every animal that is treated
- Yes, one/few randomly selected
- Yes, the largest/one of the largest
- Yes, a middle-sized one
- Yes, one of the smallest
- No

Are cows with calves and young animals (defined as animals <24 months) co-pastured?

- Yes
- No

What type of pasture is used for the animals? If several types are used, choose the alternative for the majority of the pasture period and most of the animals in 2017. Give only one answer

- Home pasture, cultivated
- Home pasture, not cultivated or harvested
- Rangeland – only together with animals from the same herd
- Rangeland - together with animals from other herds
- Other ______________________________

When are the animals usually released onto pasture? Give only one answer

| ❑ | January |
| --- | --- |
| ❑ | February |
| ❑ | March |
| ❑ | April |
| ❑ | May |
| ❑ | June |
| ❑ | July |
| ❑ | August |
| ❑ | September |
| ❑ | October |
| ❑ | November |
| ❑ | December |
| ❑ | Animals are pastured all year |

For how many months are the animals pastured every year? Answer ___________

**How well do the following sentences describe pasture use for your herd? On a scale of 1 to 5, where 1 = no agreement and 5= total agreement, cross off the most appropriate box – give only one answer per question.**

|  | 1 | | 2 | | 3 | | 4 | | 5 | |  |  |
| --- | --- | --- | --- | --- | --- | --- | --- | --- | --- | --- | --- | --- |
| Pastures are used that were not used for grazing cattle during the preceding year | | ❑ | | ❑ | | ❑ | | ❑ | | ❑ | |  |
| The same pasture is used throughout the grazing season | ❑ | | ❑ | | ❑ | | ❑ | | ❑ | |  |  |
| The animals are moved between several pastures during the course of the pasture season | ❑ | | ❑ | | ❑ | | ❑ | | ❑ | |  |  |
| Animals are released late to pasture in the spring to reduce the risk of problems with parasites | ❑ | | ❑ | | ❑ | | ❑ | | ❑ | |  |  |
| First-season grazing calves/young animals are let onto the same pasture every year | ❑ | | ❑ | | ❑ | | ❑ | | ❑ | |  |  |
| In the course of the pasture season, cattle and other animal species (e.g., horses, small ruminants) co-graze on the same pasture simultaneously | ❑ | | ❑ | | ❑ | | ❑ | | ❑ | |  |  |
| In the course of the pasture season, cattle and other animal species (e.g., horses, small ruminants) graze on the same pasture, but not simultaneously | ❑ | | ❑ | | ❑ | | ❑ | | ❑ | |  |  |
| Each year, the animal species (e.g., cattle, sheep, horses etc) grazing a particular pasture is changed | ❑ | | ❑ | | ❑ | | ❑ | | ❑ | |  |  |
| The pasture area is not used for pasturing animals some years (e.g., the pasture is harvested) | ❑ | | ❑ | | ❑ | | ❑ | | ❑ | |  |  |
| The water supply area is moved regularly | ❑ | | ❑ | | ❑ | | ❑ | | ❑ | |  |  |
| Animals with diarrhoea or other signs of pasture parasites are moved to another pasture | ❑ | | ❑ | | ❑ | | ❑ | | ❑ | |  |  |
| Animals with diarrhoea of or other signs of pasture parasites are brought inside | ❑ | | ❑ | | ❑ | | ❑ | | ❑ | |  |  |

Are you satisfied with the calves growth on the pasture?

| Very dissatisfied=1 | 2 | 3 | 4 | Very satisfied= 5 |
| --- | --- | --- | --- | --- |
| ❑ | ❑ | ❑ | ❑ | ❑ |

Do you weigh or measure the calves so that you can determine growth on the pasture?

- Yes
- No

Daily growth of calves in the pasture period is approximately (gram/day):

__________________________________________________________________________________

Estimated daily growth of calves in the pasture period is approximately (gram/day):

Have faecal samples ever been taken for investigation for pasture parasites in your herd?

- Yes
- No

Why was the faecal sample taken?

- To evaluate the need for treatment¨
- To investigate whether parasites could be the reason for disease
- To investigate whether parasites could be the reason for poor growth on pasture?
- Other reason:________________________________________________________________

___________________________________________________________________________

What do you do if symptoms that could be caused by pasture parasites occur in the grazing period (for instance diarrhoea)? (multiple answers possible)

- Treat those animals with symptoms
- Treat animals with symptoms and other animals in the same age-group as those with symptoms
- Treat every animal on pasture (every age-group)
- Take a faecal sample to determine whether the problem is due to parasites
- Move the animals to another pasture
- No particular measures
- Other measures:______________________________________________________________

___________________________________________________________________________

Where do you obtain information/advice about measures and treatment against parasites in your cattle-herd?

– multiple answers possible

- Articles in magazines for farmers
- Internet
- Other farmers
- Veterinarians
- Advisors (for instance, advisors in abattoirs, Norwegian Agricultural Extension Service, other organizations)
- Family
- Others:_____________________________________________________________________
